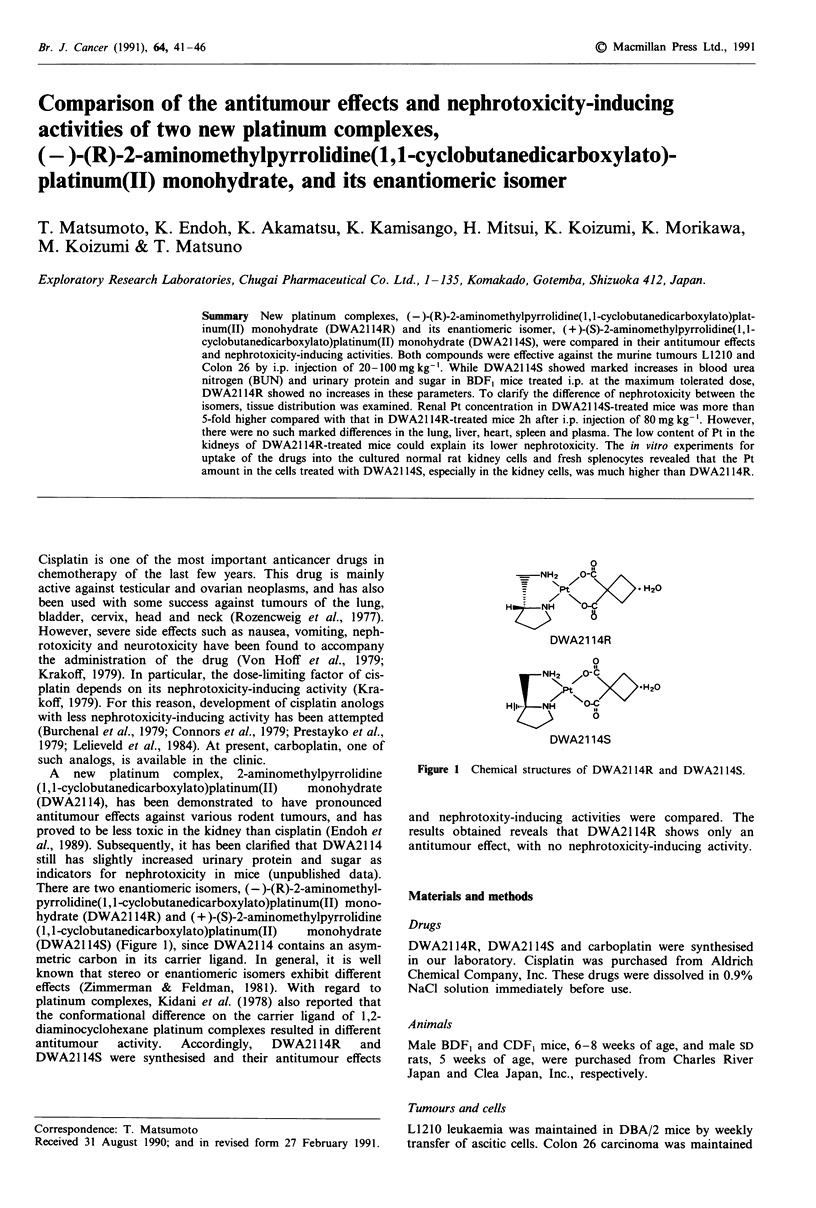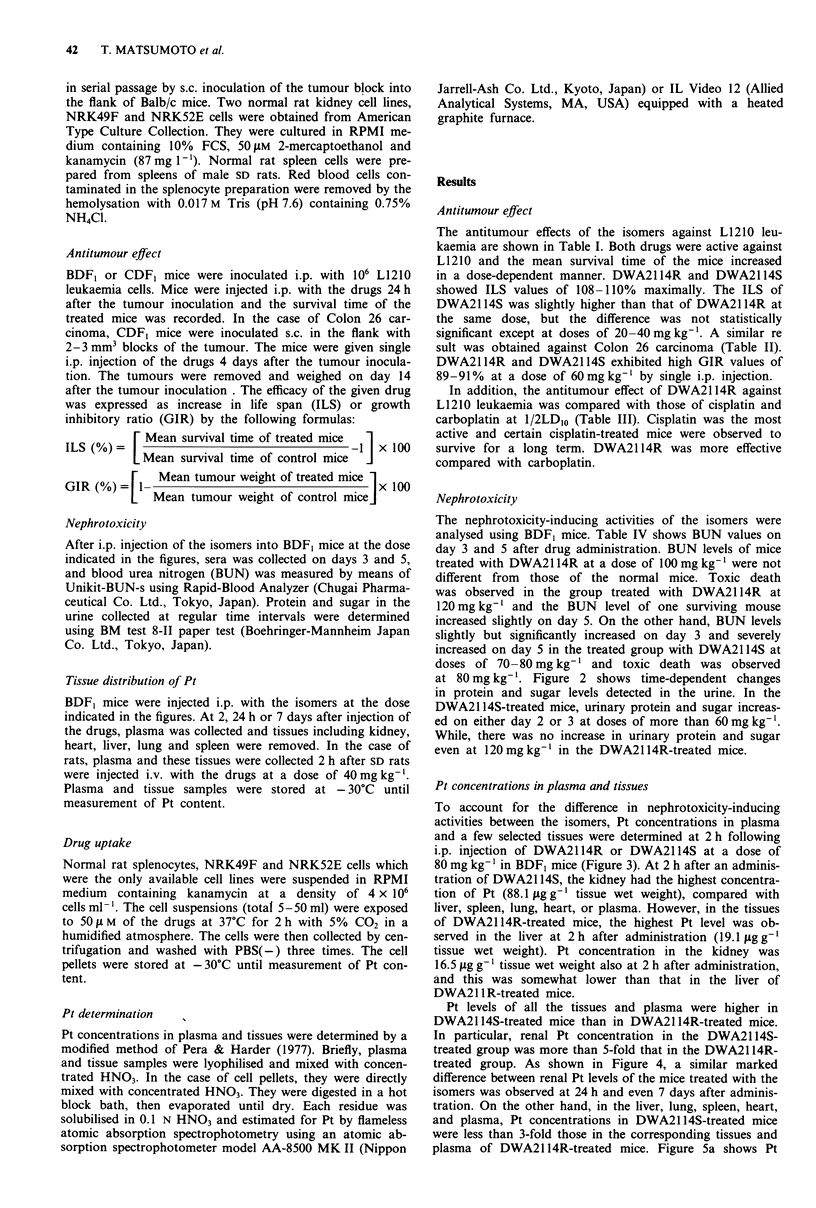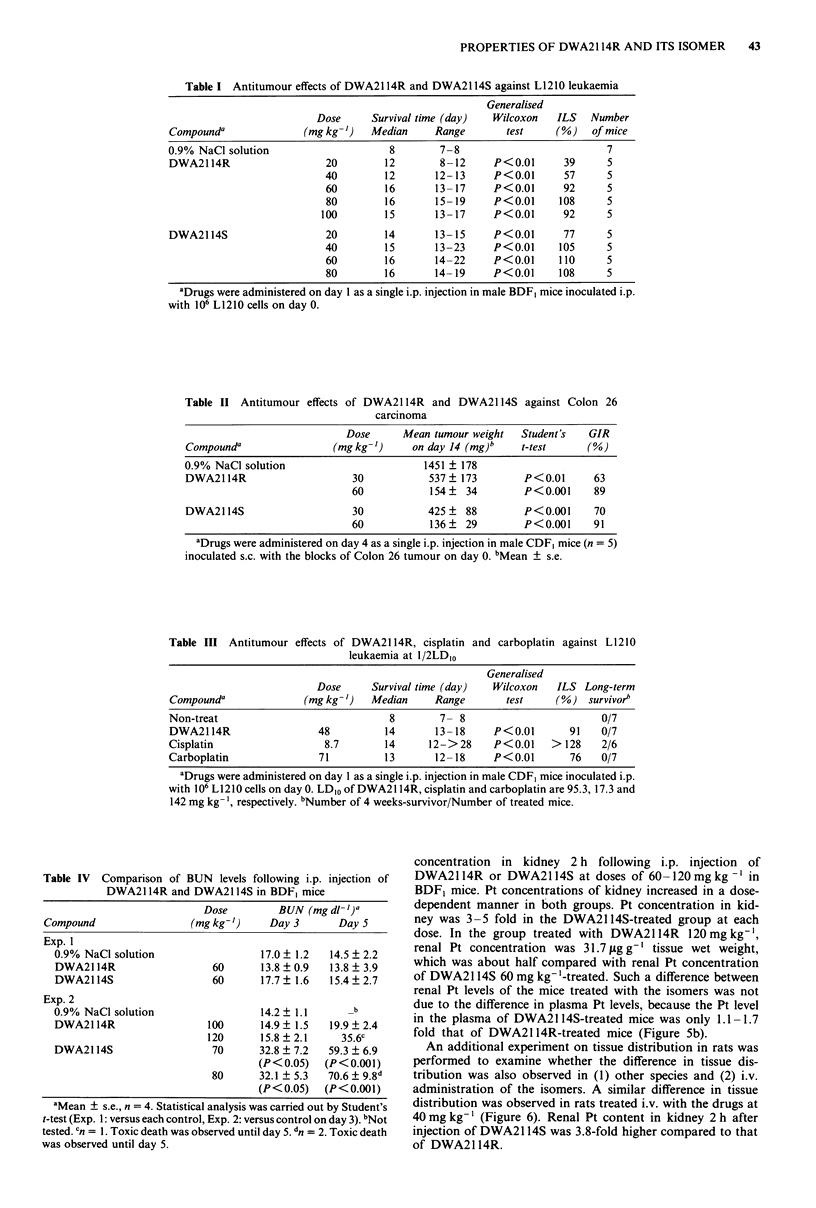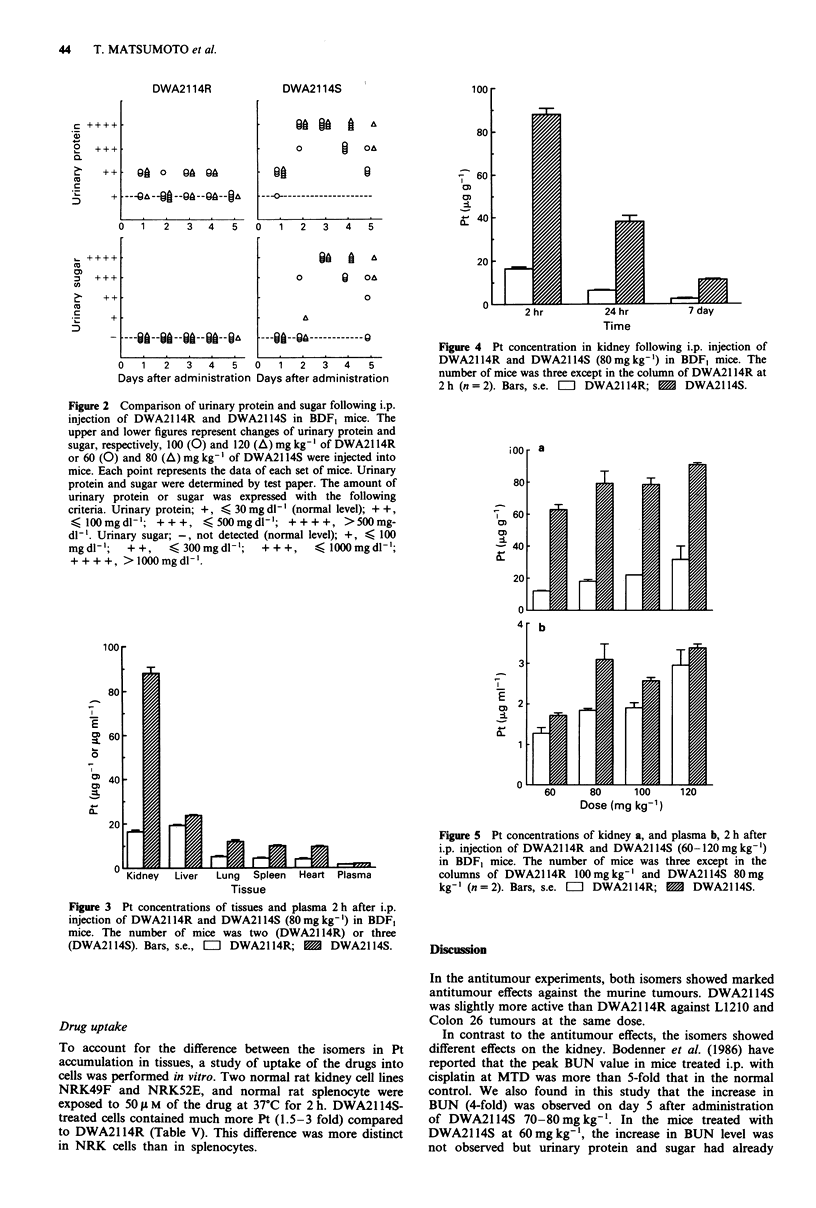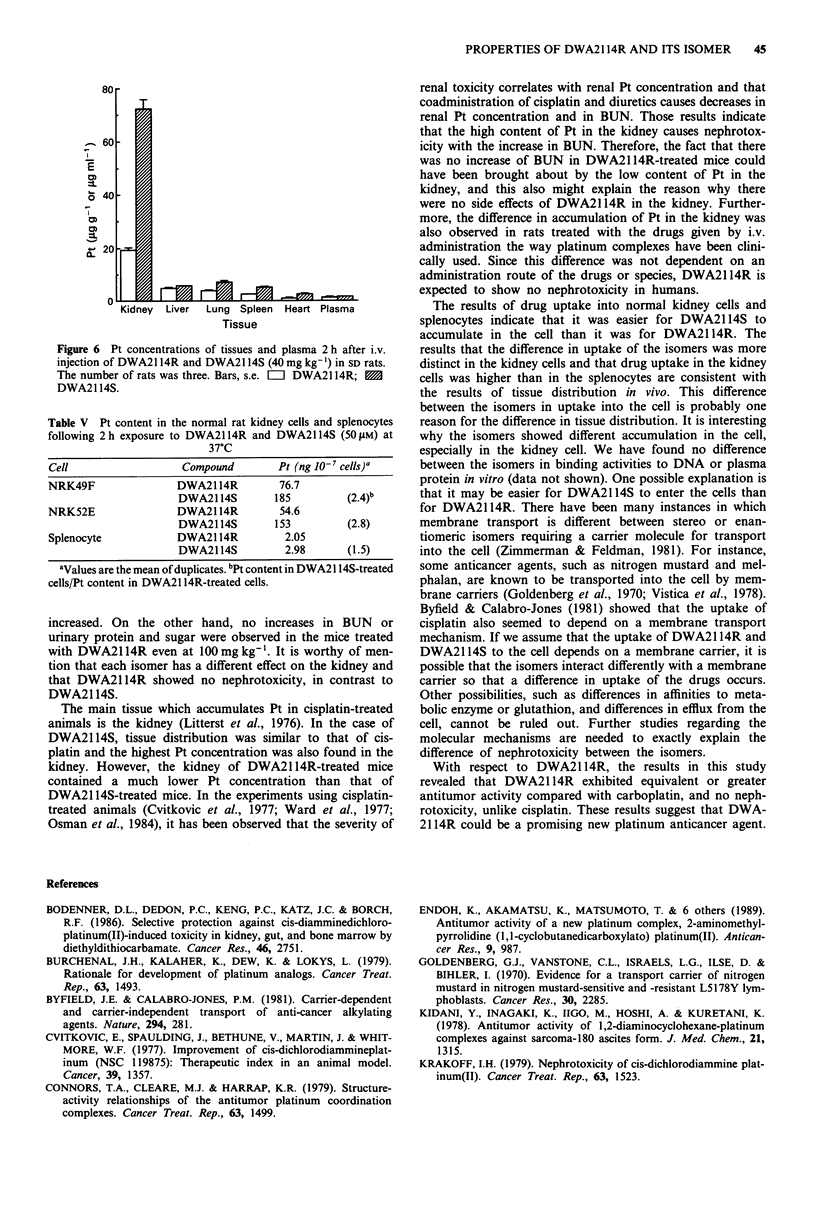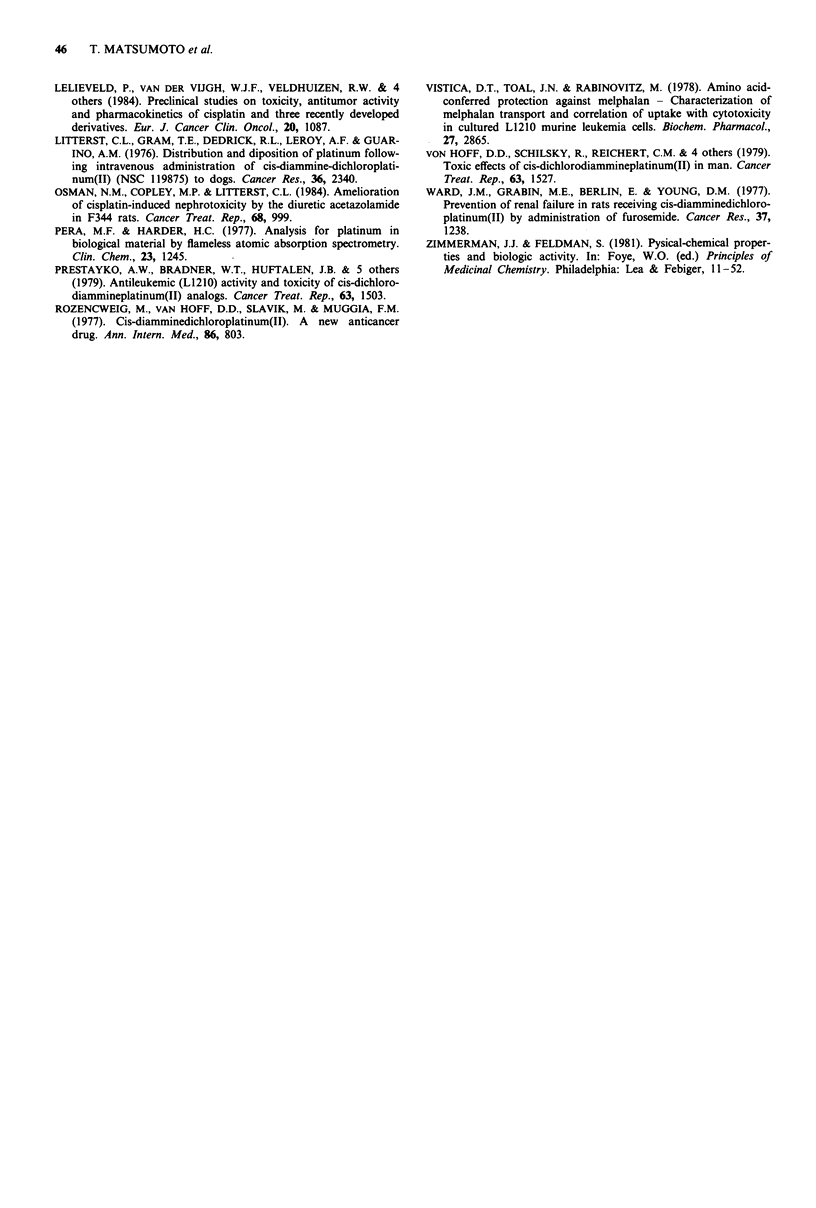# Comparison of the antitumour effects and nephrotoxicity-inducing activities of two new platinum complexes, (-)-(R)-2-aminomethylpyrrolidine(1,1-cyclobutanedicarboxylato+ ++)-platinum (II) monohydrate, and its enantiomeric isomer.

**DOI:** 10.1038/bjc.1991.236

**Published:** 1991-07

**Authors:** T. Matsumoto, K. Endoh, K. Akamatsu, K. Kamisango, H. Mitsui, K. Koizumi, K. Morikawa, M. Koizumi, T. Matsuno

**Affiliations:** Exploratory Research Laboratories, Chugai Pharmaceutical Co. Ltd., Shizuoka, Japan.

## Abstract

New platinum complexes, (-)-(R)-2-aminomethylpyrrolidine(1,1- cyclobutanedicarboxylato)platinum(II) monohydrate (DWA2114R) and its enantiomeric isomer, (+)-(S)-2-aminomethylpyrrolidine(1,1- cyclobutanedicarboxylato)platinum(II) monohydrate (DWA2114S), were compared in their antitumour effects and nephrotoxicity-inducing activities. Both compounds were effective against the murine tumours L1210 and Colon 26 by i.p. injection of 20-100 mg kg-1. While DWA2114S showed marked increases in blood urea nitrogen (BUN) and urinary protein and sugar in BDF1 mice treated i.p. at the maximum tolerated dose, DWA2114R showed no increases in these parameters. To clarify the difference of nephrotoxicity between the isomers, tissue distribution was examined. Renal Pt concentration in DWA2114S-treated mice was more than 5-fold higher compared with that in DWA2114R-treated mice 2h after i.p. injection of 80 mg kg-1. However, there were no such marked differences in the lung, liver, heart, spleen and plasma. The low content of Pt in the kidneys of DWA2114R-treated mice could explain its lower nephrotoxicity. The in vitro experiments for uptake of the drugs into the cultured normal rat kidney cells and fresh splenocytes revealed that the Pt amount in the cells treated with DWA2114S, especially in the kidney cells, was much higher than DWA2114R.


*Br. J. Cancer* (1991), **64**, 41–46 © Macmillan Press Ltd., 1991

### Comparison of the antitumour effects and nephrotoxicity-inducing activities of two new platinum complexes, (–)-(R)-2-aminomethylpyrrolidine(1,1-cyclobutanedicarboxylato)-platinum(II) monohydrate, and its enantiomeric isomer

T. Matsumoto, K. Endoh, K. Akamatsu, K. Kamisango, H. Mitsui, K. Koizumi, K. Morikawa, M. Koizumi & T. Matsuno

*Exploratory Research Laboratories, Chugai Pharmaceutical Co. Ltd., 1–135, Komakado, Gotemba, Shizuoka 412, Japan.*

**Summary** New platinum complexes, (–)-(R)-2-aminomethylpyrrolidine(1,1-cyclobutanedicarboxylato)platinum(II) monohydrate (DWA2114R) and its enantiomeric isomer, (+)-(S)-2-aminomethylpyrrolidine(1,1-cyclobutanedicarboxylato)platinum(II) monohydrate (DWA2114S), were compared in their antitumour effects and nephrotoxicity-inducing activities. Both compounds were effective against the murine tumours L1210 and Colon 26 by i.p. injection of 20–100 mg $kg^{-1}$. While DWA2114S showed marked increases in blood urea nitrogen (BUN) and urinary protein and sugar in $BDF_1$ mice treated i.p. at the maximum tolerated dose, DWA2114R showed no increases in these parameters. To clarify the difference of nephrotoxicity between the isomers, tissue distribution was examined. Renal Pt concentration in DWA2114S-treated mice was more than 5-fold higher compared with that in DWA2114R-treated mice 2h after i.p. injection of 80 mg $kg^{-1}$. However, there were no such marked differences in the lung, liver, heart, spleen and plasma. The low content of Pt in the kidneys of DWA2114R-treated mice could explain its lower nephrotoxicity. The *in vitro* experiments for uptake of the drugs into the cultured normal rat kidney cells and fresh splenocytes revealed that the Pt amount in the cells treated with DWA2114S, especially in the kidney cells, was much higher than DWA2114R.

Cisplatin is one of the most important anticancer drugs in chemotherapy of the last few years. This drug is mainly active against testicular and ovarian neoplasms, and has also been used with some success against tumours of the lung, bladder, cervix, head and neck (Rozencweig *et al.*, 1977). However, severe side effects such as nausea, vomiting, nephrotoxicity and neurotoxicity have been found to accompany the administration of the drug (Von Hoff *et al.*, 1979; Krakoff, 1979). In particular, the dose-limiting factor of cisplatin depends on its nephrotoxicity-inducing activity (Krakoff, 1979). For this reason, development of cisplatin anologs with less nephrotoxicity-inducing activity has been attempted (Burchenal *et al.*, 1979; Connors *et al.*, 1979; Prestayko *et al.*, 1979; Lelieveld *et al.*, 1984). At present, carboplatin, one of such analogs, is available in the clinic.

A new platinum complex, 2-aminomethylpyrrolidine (1,1-cyclobutanedicarboxylato)platinum(II) monohydrate (DWA2114), has been demonstrated to have pronounced antitumour effects against various rodent tumours, and has proved to be less toxic in the kidney than cisplatin (Endoh *et al.*, 1989). Subsequently, it has been clarified that DWA2114 still has slightly increased urinary protein and sugar as indicators for nephrotoxicity in mice (unpublished data). There are two enantiomeric isomers, (–)-(R)-2-aminomethylpyrrolidine(1,1-cyclobutanedicarboxylato)platinum(II) monohydrate (DWA2114R) and (+)-(S)-2-aminomethylpyrrolidine (1,1-cyclobutanedicarboxylato)platinum(II) monohydrate (DWA2114S) (Figure 1), since DWA2114 contains an asymmetric carbon in its carrier ligand. In general, it is well known that stereo or enantiomeric isomers exhibit different effects (Zimmerman & Feldman, 1981). With regard to platinum complexes, Kidani *et al.* (1978) also reported that the conformational difference on the carrier ligand of 1,2-diaminocyclohexane platinum complexes resulted in different antitumour activity. Accordingly, DWA2114R and DWA2114S were synthesised and their antitumour effects

**Figure 1** Chemical structures of DWA2114R and DWA2114S.

and nephrotoxity-inducing activities were compared. The results obtained reveals that DWA2114R shows only an antitumour effect, with no nephrotoxicity-inducing activity.

### Materials and methods

### *Drugs*

DWA2114R, DWA2114S and carboplatin were synthesised in our laboratory. Cisplatin was purchased from Aldrich Chemical Company, Inc. These drugs were dissolved in 0.9% NaCl solution immediately before use.

### *Animals*

Male $BDF_1$ and $CDF_1$ mice, 6–8 weeks of age, and male SD rats, 5 weeks of age, were purchased from Charles River Japan and Clea Japan, Inc., respectively.

### *Tumours and cells*

L1210 leukaemia was maintained in DBA/2 mice by weekly transfer of ascitic cells. Colon 26 carcinoma was maintained

Correspondence: T. Matsumoto
Received 31 August 1990; and in revised form 27 February 1991.

in serial passage by s.c. inoculation of the tumour block into the flank of Balb/c mice. Two normal rat kidney cell lines, NRK49F and NRK52E cells were obtained from American Type Culture Collection. They were cultured in RPMI medium containing 10% FCS, 50 μM 2-mercaptoethanol and kanamycin (87 mg $l^{-1}$). Normal rat spleen cells were prepared from spleens of male SD rats. Red blood cells contaminated in the splenocyte preparation were removed by the hemolysation with 0.017 M Tris (pH 7.6) containing 0.75% $NH_4Cl$.

*Antitumour effect*

$BDF_1$ or $CDF_1$ mice were inoculated i.p. with $10^6$ L1210 leukaemia cells. Mice were injected i.p. with the drugs 24 h after the tumour inoculation and the survival time of the treated mice was recorded. In the case of Colon 26 carcinoma, $CDF_1$ mice were inoculated s.c. in the flank with 2–3 $mm^3$ blocks of the tumour. The mice were given single i.p. injection of the drugs 4 days after the tumour inoculation. The tumours were removed and weighed on day 14 after the tumour inoculation . The efficacy of the given drug was expressed as increase in life span (ILS) or growth inhibitory ratio (GIR) by the following formulas:

$$\text{ILS }(\%) = \left[\frac{\text{Mean survival time of treated mice}}{\text{Mean survival time of control mice}} - 1\right] \times 100$$

$$\text{GIR }(\%) = \left[1 - \frac{\text{Mean tumour weight of treated mice}}{\text{Mean tumour weight of control mice}}\right] \times 100$$

*Nephrotoxicity*

After i.p. injection of the isomers into $BDF_1$ mice at the dose indicated in the figures, sera was collected on days 3 and 5, and blood urea nitrogen (BUN) was measured by means of Unikit-BUN-s using Rapid-Blood Analyzer (Chugai Pharmaceutical Co. Ltd., Tokyo, Japan). Protein and sugar in the urine collected at regular time intervals were determined using BM test 8-II paper test (Boehringer-Mannheim Japan Co. Ltd., Tokyo, Japan).

*Tissue distribution of Pt*

$BDF_1$ mice were injected i.p. with the isomers at the dose indicated in the figures. At 2, 24 h or 7 days after injection of the drugs, plasma was collected and tissues including kidney, heart, liver, lung and spleen were removed. In the case of rats, plasma and these tissues were collected 2 h after SD rats were injected i.v. with the drugs at a dose of 40 mg $kg^{-1}$. Plasma and tissue samples were stored at −30°C until measurement of Pt content.

*Drug uptake*

Normal rat splenocytes, NRK49F and NRK52E cells which were the only available cell lines were suspended in RPMI medium containing kanamycin at a density of $4 \times 10^6$ cells $ml^{-1}$. The cell suspensions (total 5–50 ml) were exposed to 50 μM of the drugs at 37°C for 2 h with 5% $CO_2$ in a humidified atmosphere. The cells were then collected by centrifugation and washed with PBS(−) three times. The cell pellets were stored at −30°C until measurement of Pt content.

*Pt determination*

Pt concentrations in plasma and tissues were determined by a modified method of Pera & Harder (1977). Briefly, plasma and tissue samples were lyophilised and mixed with concentrated $HNO_3$. In the case of cell pellets, they were directly mixed with concentrated $HNO_3$. They were digested in a hot block bath, then evaporated until dry. Each residue was solubilised in 0.1 N $HNO_3$ and estimated for Pt by flameless atomic absorption spectrophotometry using an atomic absorption spectrophotometer model AA-8500 MK II (Nippon Jarrell-Ash Co. Ltd., Kyoto, Japan) or IL Video 12 (Allied Analytical Systems, MA, USA) equipped with a heated graphite furnace.

### Results

*Antitumour effect*

The antitumour effects of the isomers against L1210 leukaemia are shown in Table I. Both drugs were active against L1210 and the mean survival time of the mice increased in a dose-dependent manner. DWA2114R and DWA2114S showed ILS values of 108–110% maximally. The ILS of DWA2114S was slightly higher than that of DWA2114R at the same dose, but the difference was not statistically significant except at doses of 20–40 mg $kg^{-1}$. A similar re sult was obtained against Colon 26 carcinoma (Table II). DWA2114R and DWA2114S exhibited high GIR values of 89–91% at a dose of 60 mg $kg^{-1}$ by single i.p. injection.

In addition, the antitumour effect of DWA2114R against L1210 leukaemia was compared with those of cisplatin and carboplatin at $1/2LD_{10}$ (Table III). Cisplatin was the most active and certain cisplatin-treated mice were observed to survive for a long term. DWA2114R was more effective compared with carboplatin.

*Nephrotoxicity*

The nephrotoxicity-inducing activities of the isomers were analysed using $BDF_1$ mice. Table IV shows BUN values on day 3 and 5 after drug administration. BUN levels of mice treated with DWA2114R at a dose of 100 mg $kg^{-1}$ were not different from those of the normal mice. Toxic death was observed in the group treated with DWA2114R at 120 mg $kg^{-1}$ and the BUN level of one surviving mouse increased slightly on day 5. On the other hand, BUN levels slightly but significantly increased on day 3 and severely increased on day 5 in the treated group with DWA2114S at doses of 70–80 mg $kg^{-1}$ and toxic death was observed at 80 mg $kg^{-1}$. Figure 2 shows time-dependent changes in protein and sugar levels detected in the urine. In the DWA2114S-treated mice, urinary protein and sugar increased on either day 2 or 3 at doses of more than 60 mg $kg^{-1}$. While, there was no increase in urinary protein and sugar even at 120 mg $kg^{-1}$ in the DWA2114R-treated mice.

*Pt concentrations in plasma and tissues*

To account for the difference in nephrotoxicity-inducing activities between the isomers, Pt concentrations in plasma and a few selected tissues were determined at 2 h following i.p. injection of DWA2114R or DWA2114S at a dose of 80 mg $kg^{-1}$ in $BDF_1$ mice (Figure 3). At 2 h after an administration of DWA2114S, the kidney had the highest concentration of Pt (88.1 μg $g^{-1}$ tissue wet weight), compared with liver, spleen, lung, heart, or plasma. However, in the tissues of DWA2114R-treated mice, the highest Pt level was observed in the liver at 2 h after administration (19.1 μg $g^{-1}$ tissue wet weight). Pt concentration in the kidney was 16.5 μg $g^{-1}$ tissue wet weight also at 2 h after administration, and this was somewhat lower than that in the liver of DWA211R-treated mice.

Pt levels of all the tissues and plasma were higher in DWA2114S-treated mice than in DWA2114R-treated mice. In particular, renal Pt concentration in the DWA2114S-treated group was more than 5-fold that in the DWA2114R-treated group. As shown in Figure 4, a similar marked difference between renal Pt levels of the mice treated with the isomers was observed at 24 h and even 7 days after administration. On the other hand, in the liver, lung, spleen, heart, and plasma, Pt concentrations in DWA2114S-treated mice were less than 3-fold those in the corresponding tissues and plasma of DWA2114R-treated mice. Figure 5a shows Pt

**Table I** Antitumour effects of DWA2114R and DWA2114S against L1210 leukaemia

| *Compound*[a] | *Dose (mg kg$^{-1}$)* | *Survival time (day) Median* | *Range* | *Generalised Wilcoxon test* | *ILS (%)* | *Number of mice* |
|---|---|---|---|---|---|---|
| 0.9% NaCl solution | | 8 | 7–8 | | | 7 |
| DWA2114R | 20 | 12 | 8–12 | $P<0.01$ | 39 | 5 |
| | 40 | 12 | 12–13 | $P<0.01$ | 57 | 5 |
| | 60 | 16 | 13–17 | $P<0.01$ | 92 | 5 |
| | 80 | 16 | 15–19 | $P<0.01$ | 108 | 5 |
| | 100 | 15 | 13–17 | $P<0.01$ | 92 | 5 |
| DWA2114S | 20 | 14 | 13–15 | $P<0.01$ | 77 | 5 |
| | 40 | 15 | 13–23 | $P<0.01$ | 105 | 5 |
| | 60 | 16 | 14–22 | $P<0.01$ | 110 | 5 |
| | 80 | 16 | 14–19 | $P<0.01$ | 108 | 5 |

[a]Drugs were administered on day 1 as a single i.p. injection in male $BDF_1$ mice inoculated i.p. with $10^6$ L1210 cells on day 0.

**Table II** Antitumour effects of DWA2114R and DWA2114S against Colon 26 carcinoma

| *Compound*[a] | *Dose (mg kg$^{-1}$)* | *Mean tumour weight on day 14 (mg)*[b] | *Student's t-test* | *GIR (%)* |
|---|---|---|---|---|
| 0.9% NaCl solution | | 1451 ± 178 | | |
| DWA2114R | 30 | 537 ± 173 | $P<0.01$ | 63 |
| | 60 | 154 ± 34 | $P<0.001$ | 89 |
| DWA2114S | 30 | 425 ± 88 | $P<0.001$ | 70 |
| | 60 | 136 ± 29 | $P<0.001$ | 91 |

[a]Drugs were administered on day 4 as a single i.p. injection in male $CDF_1$ mice ($n = 5$) inoculated s.c. with the blocks of Colon 26 tumour on day 0. [b]Mean ± s.e.

**Table III** Antitumour effects of DWA2114R, cisplatin and carboplatin against L1210 leukaemia at $1/2LD_{10}$

| *Compound*[a] | *Dose (mg kg$^{-1}$)* | *Survival time (day) Median* | *Range* | *Generalised Wilcoxon test* | *ILS (%)* | *Long-term survivor*[b] |
|---|---|---|---|---|---|---|
| Non-treat | | 8 | 7– 8 | | | 0/7 |
| DWA2114R | 48 | 14 | 13–18 | $P<0.01$ | 91 | 0/7 |
| Cisplatin | 8.7 | 14 | 12–>28 | $P<0.01$ | >128 | 2/6 |
| Carboplatin | 71 | 13 | 12–18 | $P<0.01$ | 76 | 0/7 |

[a]Drugs were administered on day 1 as a single i.p. injection in male $CDF_1$ mice inoculated i.p. with $10^6$ L1210 cells on day 0. $LD_{10}$ of DWA2114R, cisplatin and carboplatin are 95.3, 17.3 and 142 mg kg$^{-1}$, respectively. [b]Number of 4 weeks-survivor/Number of treated mice.

**Table IV** Comparison of BUN levels following i.p. injection of DWA2114R and DWA2114S in $BDF_1$ mice

| *Compound* | *Dose (mg kg$^{-1}$)* | *BUN (mg dl$^{-1}$)*[a] *Day 3* | *Day 5* |
|---|---|---|---|
| Exp. 1 | | | |
| 0.9% NaCl solution | | 17.0 ± 1.2 | 14.5 ± 2.2 |
| DWA2114R | 60 | 13.8 ± 0.9 | 13.8 ± 3.9 |
| DWA2114S | 60 | 17.7 ± 1.6 | 15.4 ± 2.7 |
| Exp. 2 | | | |
| 0.9% NaCl solution | | 14.2 ± 1.1 | –[b] |
| DWA2114R | 100 | 14.9 ± 1.5 | 19.9 ± 2.4 |
| | 120 | 15.8 ± 2.1 | 35.6[c] |
| DWA2114S | 70 | 32.8 ± 7.2 ($P<0.05$) | 59.3 ± 6.9 ($P<0.001$) |
| | 80 | 32.1 ± 5.3 ($P<0.05$) | 70.6 ± 9.8[d] ($P<0.001$) |

[a]Mean ± s.e., $n = 4$. Statistical analysis was carried out by Student's $t$-test (Exp. 1: versus each control, Exp. 2: versus control on day 3). [b]Not tested. [c]$n = 1$. Toxic death was observed until day 5. [d]$n = 2$. Toxic death was observed until day 5.

concentration in kidney 2 h following i.p. injection of DWA2114R or DWA2114S at doses of 60–120 mg kg$^{-1}$ in $BDF_1$ mice. Pt concentrations of kidney increased in a dose-dependent manner in both groups. Pt concentration in kidney was 3–5 fold in the DWA2114S-treated group at each dose. In the group treated with DWA2114R 120 mg kg$^{-1}$, renal Pt concentration was 31.7 μg g$^{-1}$ tissue wet weight, which was about half compared with renal Pt concentration of DWA2114S 60 mg kg$^{-1}$-treated. Such a difference between renal Pt levels of the mice treated with the isomers was not due to the difference in plasma Pt levels, because the Pt level in the plasma of DWA2114S-treated mice was only 1.1–1.7 fold that of DWA2114R-treated mice (Figure 5b).

An additional experiment on tissue distribution in rats was performed to examine whether the difference in tissue distribution was also observed in (1) other species and (2) i.v. administration of the isomers. A similar difference in tissue distribution was observed in rats treated i.v. with the drugs at 40 mg kg$^{-1}$ (Figure 6). Renal Pt content in kidney 2 h after injection of DWA2114S was 3.8-fold higher compared to that of DWA2114R.

**Figure 2** Comparison of urinary protein and sugar following i.p. injection of DWA2114R and DWA2114S in $BDF_1$ mice. The upper and lower figures represent changes of urinary protein and sugar, respectively, 100 (O) and 120 (Δ) mg $kg^{-1}$ of DWA2114R or 60 (O) and 80 (Δ) mg $kg^{-1}$ of DWA2114S were injected into mice. Each point represents the data of each set of mice. Urinary protein and sugar were determined by test paper. The amount of urinary protein or sugar was expressed with the following criteria. Urinary protein; +, $\leqslant$ 30 mg $dl^{-1}$ (normal level); ++, $\leqslant$ 100 mg $dl^{-1}$; +++, $\leqslant$ 500 mg $dl^{-1}$; ++++, > 500 mg $dl^{-1}$. Urinary sugar; −, not detected (normal level); +, $\leqslant$ 100 mg $dl^{-1}$; ++, $\leqslant$ 300 mg $dl^{-1}$; +++, $\leqslant$ 1000 mg $dl^{-1}$; ++++, > 1000 mg $dl^{-1}$.

**Figure 3** Pt concentrations of tissues and plasma 2 h after i.p. injection of DWA2114R and DWA2114S (80 mg $kg^{-1}$) in $BDF_1$ mice. The number of mice was two (DWA2114R) or three (DWA2114S). Bars, s.e., ☐ DWA2114R; ▨ DWA2114S.

### Drug uptake

To account for the difference between the isomers in Pt accumulation in tissues, a study of uptake of the drugs into cells was performed *in vitro*. Two normal rat kidney cell lines NRK49F and NRK52E, and normal rat splenocyte were exposed to 50 µM of the drug at 37°C for 2 h. DWA2114S-treated cells contained much more Pt (1.5–3 fold) compared to DWA2114R (Table V). This difference was more distinct in NRK cells than in splenocytes.

**Figure 4** Pt concentration in kidney following i.p. injection of DWA2114R and DWA2114S (80 mg $kg^{-1}$) in $BDF_1$ mice. The number of mice was three except in the column of DWA2114R at 2 h ($n = 2$). Bars, s.e. ☐ DWA2114R; ▨ DWA2114S.

**Figure 5** Pt concentrations of kidney **a**, and plasma **b**, 2 h after i.p. injection of DWA2114R and DWA2114S (60–120 mg $kg^{-1}$) in $BDF_1$ mice. The number of mice was three except in the columns of DWA2114R 100 mg $kg^{-1}$ and DWA2114S 80 mg $kg^{-1}$ ($n = 2$). Bars, s.e. ☐ DWA2114R; ▨ DWA2114S.

### Discussion

In the antitumour experiments, both isomers showed marked antitumour effects against the murine tumours. DWA2114S was slightly more active than DWA2114R against L1210 and Colon 26 tumours at the same dose.

In contrast to the antitumour effects, the isomers showed different effects on the kidney. Bodenner *et al.* (1986) have reported that the peak BUN value in mice treated i.p. with cisplatin at MTD was more than 5-fold that in the normal control. We also found in this study that the increase in BUN (4-fold) was observed on day 5 after administration of DWA2114S 70–80 mg $kg^{-1}$. In the mice treated with DWA2114S at 60 mg $kg^{-1}$, the increase in BUN level was not observed but urinary protein and sugar had already

**Figure 6** Pt concentrations of tissues and plasma 2 h after i.v. injection of DWA2114R and DWA2114S (40 mg $kg^{-1}$) in SD rats. The number of rats was three. Bars, s.e. ☐ DWA2114R; ▨ DWA2114S.

**Table V** Pt content in the normal rat kidney cells and splenocytes following 2 h exposure to DWA2114R and DWA2114S (50 μM) at 37°C

| *Cell* | *Compound* | *Pt (ng $10^{-7}$ cells)*[a] | |
|---|---|---|---|
| NRK49F | DWA2114R | 76.7 | |
| | DWA2114S | 185 | (2.4)[b] |
| NRK52E | DWA2114R | 54.6 | |
| | DWA2114S | 153 | (2.8) |
| Splenocyte | DWA2114R | 2.05 | |
| | DWA2114S | 2.98 | (1.5) |

[a]Values are the mean of duplicates. [b]Pt content in DWA2114S-treated cells/Pt content in DWA2114R-treated cells.

increased. On the other hand, no increases in BUN or urinary protein and sugar were observed in the mice treated with DWA2114R even at 100 mg $kg^{-1}$. It is worthy of mention that each isomer has a different effect on the kidney and that DWA2114R showed no nephrotoxicity, in contrast to DWA2114S.

The main tissue which accumulates Pt in cisplatin-treated animals is the kidney (Litterst *et al.*, 1976). In the case of DWA2114S, tissue distribution was similar to that of cisplatin and the highest Pt concentration was also found in the kidney. However, the kidney of DWA2114R-treated mice contained a much lower Pt concentration than that of DWA2114S-treated mice. In the experiments using cisplatin-treated animals (Cvitkovic *et al.*, 1977; Ward *et al.*, 1977; Osman *et al.*, 1984), it has been observed that the severity of renal toxicity correlates with renal Pt concentration and that coadministration of cisplatin and diuretics causes decreases in renal Pt concentration and in BUN. Those results indicate that the high content of Pt in the kidney causes nephrotoxicity with the increase in BUN. Therefore, the fact that there was no increase of BUN in DWA2114R-treated mice could have been brought about by the low content of Pt in the kidney, and this also might explain the reason why there were no side effects of DWA2114R in the kidney. Furthermore, the difference in accumulation of Pt in the kidney was also observed in rats treated with the drugs given by i.v. administration the way platinum complexes have been clinically used. Since this difference was not dependent on an administration route of the drugs or species, DWA2114R is expected to show no nephrotoxicity in humans.

The results of drug uptake into normal kidney cells and splenocytes indicate that it was easier for DWA2114S to accumulate in the cell than it was for DWA2114R. The results that the difference in uptake of the isomers was more distinct in the kidney cells and that drug uptake in the kidney cells was higher than in the splenocytes are consistent with the results of tissue distribution *in vivo*. This difference between the isomers in uptake into the cell is probably one reason for the difference in tissue distribution. It is interesting why the isomers showed different accumulation in the cell, especially in the kidney cell. We have found no difference between the isomers in binding activities to DNA or plasma protein *in vitro* (data not shown). One possible explanation is that it may be easier for DWA2114S to enter the cells than for DWA2114R. There have been many instances in which membrane transport is different between stereo or enantiomeric isomers requiring a carrier molecule for transport into the cell (Zimmerman & Feldman, 1981). For instance, some anticancer agents, such as nitrogen mustard and melphalan, are known to be transported into the cell by membrane carriers (Goldenberg *et al.*, 1970; Vistica *et al.*, 1978). Byfield & Calabro-Jones (1981) showed that the uptake of cisplatin also seemed to depend on a membrane transport mechanism. If we assume that the uptake of DWA2114R and DWA2114S to the cell depends on a membrane carrier, it is possible that the isomers interact differently with a membrane carrier so that a difference in uptake of the drugs occurs. Other possibilities, such as differences in affinities to metabolic enzyme or glutathion, and differences in efflux from the cell, cannot be ruled out. Further studies regarding the molecular mechanisms are needed to exactly explain the difference of nephrotoxicity between the isomers.

With respect to DWA2114R, the results in this study revealed that DWA2114R exhibited equivalent or greater antitumor activity compared with carboplatin, and no nephrotoxicity, unlike cisplatin. These results suggest that DWA-2114R could be a promising new platinum anticancer agent.

### References

BODENNER, D.L., DEDON, P.C., KENG, P.C., KATZ, J.C. & BORCH, R.F. (1986). Selective protection against cis-diamminedichloroplatinum(II)-induced toxicity in kidney, gut, and bone marrow by diethyldithiocarbamate. *Cancer Res.*, **46**, 2751.

BURCHENAL, J.H., KALAHER, K., DEW, K. & LOKYS, L. (1979). Rationale for development of platinum analogs. *Cancer Treat. Rep.*, **63**, 1493.

BYFIELD, J.E. & CALABRO-JONES, P.M. (1981). Carrier-dependent and carrier-independent transport of anti-cancer alkylating agents. *Nature*, **294**, 281.

CVITKOVIC, E., SPAULDING, J., BETHUNE, V., MARTIN, J. & WHITMORE, W.F. (1977). Improvement of cis-dichlorodiammineplatinum (NSC 119875): Therapeutic index in an animal model. *Cancer*, **39**, 1357.

CONNORS, T.A., CLEARE, M.J. & HARRAP, K.R. (1979). Structure-activity relationships of the antitumor platinum coordination complexes. *Cancer Treat. Rep.*, **63**, 1499.

ENDOH, K., AKAMATSU, K., MATSUMOTO, T. & 6 others (1989). Antitumor activity of a new platinum complex, 2-aminomethylpyrrolidine (1,1-cyclobutanedicarboxylato) platinum(II). *Anticancer Res.*, **9**, 987.

GOLDENBERG, G.J., VANSTONE, C.L., ISRAELS, L.G., ILSE, D. & BIHLER, I. (1970). Evidence for a transport carrier of nitrogen mustard in nitrogen mustard-sensitive and -resistant L5178Y lymphoblasts. *Cancer Res.*, **30**, 2285.

KIDANI, Y., INAGAKI, K., IIGO, M., HOSHI, A. & KURETANI, K. (1978). Antitumor activity of 1,2-diaminocyclohexane-platinum complexes against sarcoma-180 ascites form. *J. Med. Chem.*, **21**, 1315.

KRAKOFF, I.H. (1979). Nephrotoxicity of cis-dichlorodiammine platinum(II). *Cancer Treat. Rep.*, **63**, 1523.

LELIEVELD, P., VAN DER VIJGH, W.J.F., VELDHUIZEN, R.W. & 4 others (1984). Preclinical studies on toxicity, antitumor activity and pharmacokinetics of cisplatin and three recently developed derivatives. *Eur. J. Cancer Clin. Oncol.*, **20**, 1087.

LITTERST, C.L., GRAM, T.E., DEDRICK, R.L., LEROY, A.F. & GUARINO, A.M. (1976). Distribution and diposition of platinum following intravenous administration of cis-diammine-dichloroplatinum(II) (NSC 119875) to dogs. *Cancer Res.*, **36**, 2340.

OSMAN, N.M., COPLEY, M.P. & LITTERST, C.L. (1984). Amelioration of cisplatin-induced nephrotoxicity by the diuretic acetazolamide in F344 rats. *Cancer Treat. Rep.*, **68**, 999.

PERA, M.F. & HARDER, H.C. (1977). Analysis for platinum in biological material by flameless atomic absorption spectrometry. *Clin. Chem.*, **23**, 1245.

PRESTAYKO, A.W., BRADNER, W.T., HUFTALEN, J.B. & 5 others (1979). Antileukemic (L1210) activity and toxicity of cis-dichlorodiammineplatinum(II) analogs. *Cancer Treat. Rep.*, **63**, 1503.

ROZENCWEIG, M., VAN HOFF, D.D., SLAVIK, M. & MUGGIA, F.M. (1977). Cis-diamminedichloroplatinum(II). A new anticancer drug. *Ann. Intern. Med.*, **86**, 803.

VISTICA, D.T., TOAL, J.N. & RABINOVITZ, M. (1978). Amino acid-conferred protection against melphalan – Characterization of melphalan transport and correlation of uptake with cytotoxicity in cultured L1210 murine leukemia cells. *Biochem. Pharmacol.*, **27**, 2865.

VON HOFF, D.D., SCHILSKY, R., REICHERT, C.M. & 4 others (1979). Toxic effects of cis-dichlorodiammineplatinum(II) in man. *Cancer Treat. Rep.*, **63**, 1527.

WARD, J.M., GRABIN, M.E., BERLIN, E. & YOUNG, D.M. (1977). Prevention of renal failure in rats receiving cis-diamminedichloroplatinum(II) by administration of furosemide. *Cancer Res.*, **37**, 1238.

ZIMMERMAN, J.J. & FELDMAN, S. (1981). Pysical-chemical properties and biologic activity. In: Foye, W.O. (ed.) *Principles of Medicinal Chemistry*. Philadelphia: Lea & Febiger, 11–52.